# The correlation of anterior segment structures in primary congenital glaucoma by ultrasound biomicroscopy with disease severity and surgical outcomes

**DOI:** 10.1007/s00417-023-06308-6

**Published:** 2023-11-08

**Authors:** Qingdan Xu, Youjia Zhang, Li Wang, Xueli Chen, Xinghuai Sun, Yuhong Chen

**Affiliations:** 1 Department of Ophthalmology, Eye Ear Nose and Throat Hospital, Fudan University, 83 Fenyang Road, Shanghai, 200031 China; 2 NHC Key Laboratory of Myopia, Chinese Academy of Medical Sciences, and Shanghai Key Laboratory of Visual Impairment and Restoration, Fudan University, Shanghai, China; 3 State Key Laboratory of Medical Neurobiology, Institutes of Brain Science, Fudan University, Shanghai, China

**Keywords:** Anterior segment, Disease severity, Primary congenital glaucoma, Surgical outcome, Ultrasound biomicroscopy

## Abstract

**Purpose:**

To evaluate the anterior segment structures using ultrasound biomicroscopy (UBM) in primary congenital glaucoma (PCG) and explore their correlation with disease severity and surgical outcomes.

**Methods:**

Clinical information of PCG patients who underwent UBM prior to their first glaucoma surgeries from September 2014 to March 2021 were reviewed. The study included 214 UBM images of 154 PCG eyes and 60 fellow unaffected eyes. Anterior segment characteristics were analyzed. UBM parameters, including the iris thickness (IT) at variant distances from the pupil edge and iris root, anterior chamber depth (ACD), and pupil diameter (PD), were compared between two groups and their relationship with clinical factors and surgical outcomes were analyzed in PCG eyes.

**Results:**

PCG eyes had unclear scleral spur, thin iris, wide anterior chamber angle, deep anterior chamber, rarefied ciliary body, elongated ciliary processes, and abnormal anterior iris insertion. ITs were thinner, ACD was deeper, and PD was larger in PCG eyes than fellow unaffected eyes (all *P* < 0.001). In PCG eyes, thinner ITs correlated with bilateral involvement and earlier age at presentation, and larger PD correlated with earlier age at presentation (*P* = 0.030) and higher intraocular pressure (*P* < 0.001). Thinner IT2 (*P* = 0.046) and larger PD (*P* = 0.049) were identified as risk factors for surgical failure.

**Conclusion:**

UBM is a powerful technique to exam anterior segment structures in PCG. The anatomical features are associated with disease severity and surgical outcomes, providing essential clinical insights.

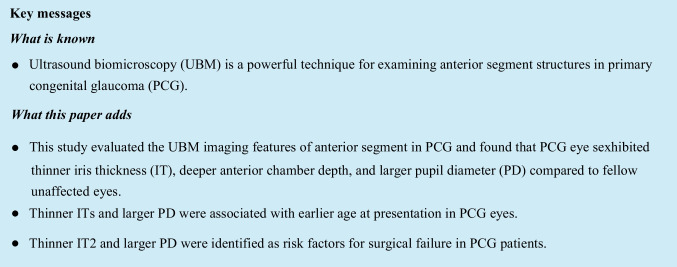

## Introduction

Primary congenital glaucoma (PCG) is the most common type of childhood glaucoma and results from the developmental defects of aqueous humor outflow structures, which leads to the increase of aqueous humor outflow resistance and elevation of intraocular pressure (IOP) [1–4]. Aside from elevated IOP, PCG is also characterized by globe enlargement (buphthalmos), corneal edema, breaks in the Descemet’s membrane (Haab striae), and progressive optic nerve damage [5]. Surgery is the main management of PCG, which can effectively control IOP and prevent further visual impairment [6, 7]. However, the surgical outcome can be unsatisfactory in some PCG cases and multiple surgeries may be required during follow-up [8–10].

Assessment of the morphology of the anterior segment structures is important for the diagnosis and surgical management of PCG [2]. However, due to the corneal edema and opacity in PCG patients, it is challenging to clearly observe the anterior segment structures by slit lamp and gonioscopy. Ultrasound biomicroscopy (UBM) is a noninvasive, dynamic, and real-time imaging technique [2, 11]. It provides in vivo visualization of the anterior segment structures and is particularly suitable for evaluation of PCG with opaque cornea [12].

Several studies have previously described the ultrasound biomicroscopic features of the anterior segment structures in PCG, including thinner iris thickness (IT), deeper anterior chamber depth (ACD), larger anterior chamber angle, thinner ciliary body thickness, longer zonular length and anterior iris insertion [2, 11–13]. However, no published study has yet established the relationship between the UBM parameters and the clinical characteristics of PCG, as well as the surgical prognosis. The purpose of the present study is to explore the correlation between the anatomical characteristics of anterior segment structures of PCG by UBM with disease severity and surgical outcomes.

## Methods

### Study design

A retrospective case-control study design was employed in this research.

### Patients

One hundred and fifty-four patients of PCG examined with UBM were enrolled from September 2014 to March 2021 in Eye Ear Nose and Throat Hospital of Fudan University. Diagnosis of PCG was based on fulfilling at least two of the following criteria: (1) IOP >21 mmHg, (2) corneal findings including enlarged corneal diameter, corneal edema or Haab striae, (3) glaucomatous optic neuropathy, (4) increased axial length (AL) exceeding normal growth [14]. Eyes with anterior segment dysgenesis, secondary glaucoma, histories of intraocular surgery or trauma were excluded in this study. Patients were followed up postoperatively for 53.5 (34–70) months. This study was approved by the Institutional Review Board of Eye Ear Nose and Throat Hospital of Fudan University (KJ2011-04) and followed the tenets of the Declaration of Helsinki. Written consent forms were obtained from all patients or their guardians.

### Clinical investigation

The medical records of all the patients were reviewed with their demographic data including gender, laterality, age at presentation, and age at examination. Each patient underwent complete ophthalmologic examinations under general anesthesia before the surgeries, including ophthalmoscopy, gonioscopy, measurement of corneal diameter using a caliper, IOP measurement using Schiotz tonometer (66 Vision Tech Co., Ltd., Suzhou, China), Goldmann applanation tonometer (Haag-Streit AG, Koeniz, Switzerland) or Tono-Pen (Reichert Technologies, Depew, NY, USA), B-mode ultrasonography (Quantel Medical, Cournon d’Auvergne Cedex, France), A-mode ultrasonography (AL-3000; Tomey Corporation, Nagoya, Japan), and UBM (MD-300L; MEDA Co., Ltd., Tianjin, China). IOP, horizontal corneal diameter (HCD), vertical cup-to-disc ratio (VCDR), and AL were recorded for analysis. Patients were followed up regularly after operation. Surgical success was defined as a postoperative IOP ≤21 mmHg without antiglaucoma medications or reoperation, based on the last recorded IOP during the postoperative follow-up. We included only patients whose postoperative follow-up exceeded 12 months in the prognosis analysis.

### UBM examination

**Fig. 1:**
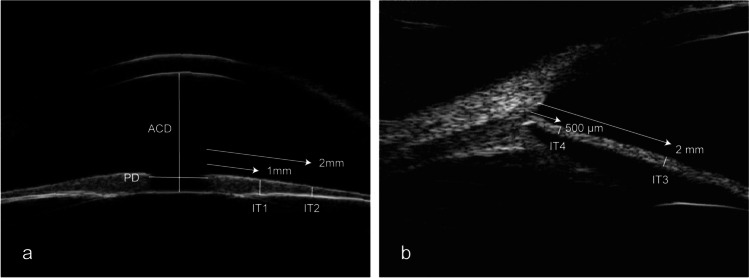
Details of measurement of the anterior chamber parameters. **a** Iris thickness 1 (IT1), iris thickness 2 (IT2), anterior chamber depth (ACD), and pupil diameter (PD). **b** Iris thickness 3 (IT3) and iris thickness 4 (IT4)

UBM examination with 50 MHz resolution was performed in supine position for biometric quantitative evaluation of the anterior segment. Details of the measurements from UBM figures are presented as follows (Fig. 1). Iris thickness 1 (IT1): measured across a vertical line perpendicular to the posterior iris plane at 1 mm from the pupil edge. Iris thickness 2 (IT2): measured across a vertical line perpendicular to the posterior iris plane at 2 mm from the pupil edge. Iris thickness 3 (IT3): measured across a vertical line perpendicular to the posterior iris plane at 2 mm from the iris root. Iris thickness 4 (IT4): measured across a vertical line perpendicular to the posterior iris plane at 500 μm from the iris root. Anterior chamber depth (ACD): measured from the center of corneal endothelium to the center of anterior lens capsule. Pupil diameter (PD): measured along the line between the pupil edge. The anterior chamber parameters were measured on each high-quality image and the average values were used for analysis.

### Statistical analysis

All data were analyzed using SPSS version 20.0 (IBM-SPSS, Chicago, IL, USA). For patients diagnosed with bilateral PCG, the more severe eyes were included in the PCG group. For those with unilateral PCG, the affected eyes were included in the PCG group and the fellow unaffected eyes were served as the control group. Student *t* test and Mann-Whitney test were used to compare the differences in quantitative variables between the PCG and control groups. Comparisons of categorical data were assessed using Pearson’s chi square test. Bivariate correlations and multivariate linear regression analysis were performed to evaluate the relationships between the UBM parameters and clinical features in the PCG group. Univariate and multivariate logistic regression analyses were used to determine the risk factors of surgical failure. Only statistically significant variables from the univariate analysis were further analyzed in the multivariate analysis. Statistical significance was set at *P* < 0.05.

## Results

### Demographic characteristics

In total, 214 eyes of 154 patients were enrolled in this study, including 154 PCG eyes as the case group and 60 fellow unaffected eyes as the control group. Ninety-four patients (61.0%) had bilateral PCG and 60 patients (39.0%) had unilateral PCG. Ninety-three patients were male (60.4%) and 61 patients were female (39.6%). The median age at presentation was 6 (1–18) months, with 103 patients (66.9%) presenting symptoms in the first year of life. All the 154 PCG eyes in the case group underwent glaucoma surgeries, of which 146 eyes (94.8%) underwent trabeculotomy, four eyes (2.6%) underwent trabeculectomy, and four eyes (2.6%) underwent combined trabeculotomy-trabeculectomy as their first operations.

### Clinical characteristics

Comparisons of clinical characteristics between the PCG group and the control group are presented in Table 1. The age at examination in the PCG group ranged between 1 and 116 months with a median of 10 months, and the age at examination in the control group ranged between 2 and 91 months with a median of 13.5 months, with no significant difference between two groups (*P* = 0.158). The PCG group had a higher IOP, larger HCD, greater VCDR, and longer AL than the control group (all *P* < 0.001).

**Table 1:** Comparison of clinical characteristics and anterior chamber parameters between groups

	PCG group	Control group	*P*
Eyes	154	60	/
Gender (male/female)	93/61	38/22	0.691
Age at examination (month), median (range)	10 (1-116)	13.5 (2-91)	0.158
IOP (mmHg), median (range)	31.3 (15.0-69.2)	14.6 (5.0-24.3)	<0.001*
HCD (mm), median (range)	13.0 (10.5-15.0)	11.5 (9.0-12.5)	<0.001*
VCDR, median (range)	0.9 (0.4-1.0)	0.4 (0.2-0.8)	<0.001*
AL (mm), median (range)	24.04 (19.85-31.52)	21.04 (17.72-23.53)	<0.001*
IT1 (mm), median (range)	0.35 (0.11-0.53)	0.44 (0.30-0.64)	<0.001*
IT2 (mm), median (range)	0.27 (0.08-0.42)	0.33 (0.19-0.46)	<0.001*
IT3 (mm), median (range)	0.21 (0.07-0.33)	0.25 (0.15-0.34)	<0.001*
IT4 (mm), median (range)	0.16 (0.07-0.23)	0.18 (0.10-0.28)	<0.001*
ACD (mm), median (range)	3.77 (2.16-5.19)	2.82 (1.49-3.42)	<0.001*
PD (mm), median (range)	2.35 (0.93-7.19)	1.75 (1.27-2.88)	<0.001*

*Statistical significance; *PCG*, primary congenital glaucoma; *IOP*, intraocular pressure; *HCD*, horizontal corneal diameter; *VCDR*, vertical cup-to-disc ratio; *AL*, axial length; *IT*, iris thickness; *ACD*, anterior chamber depth; *PD*, pupil diameter

### Characteristics of anterior segment observed by UBM in PCG

**Fig. 2:**
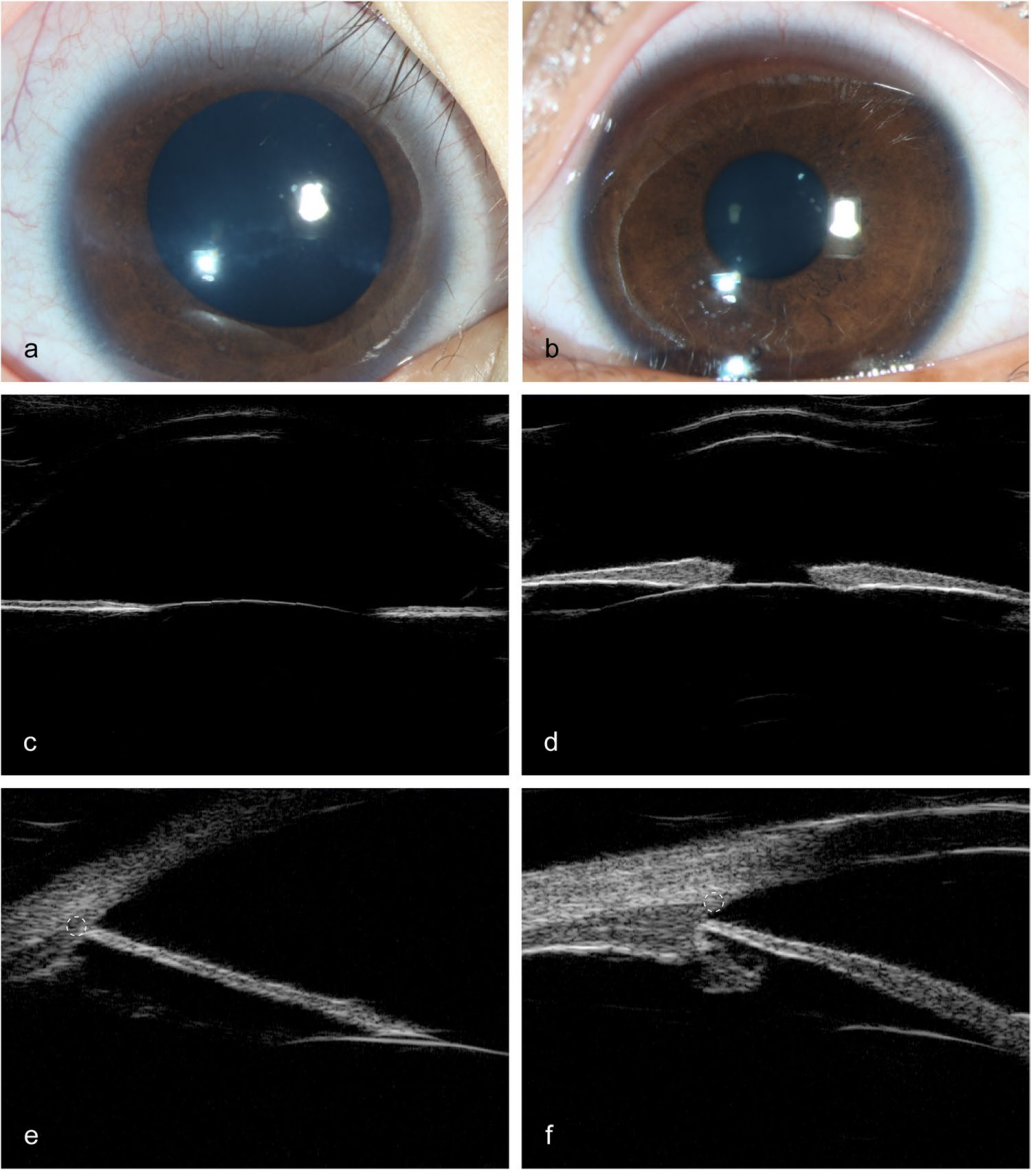
The anterior segment photographs and UBM images of a patient with unilateral PCG. **a** Slight corneal edema, Haab striae, and large pupil diameter with corectopia in the PCG eye. **b** Normal appearance of the fellow unaffected eye. **c**, **e** Thinner iris, wider anterior chamber angle, deeper anterior chamber, rarefied ciliary body, and abnormal anterior iris insertion in the PCG eye. **d**, **f** Normal-appearing anterior chamber angle of the fellow unaffected eye. The scleral spur is shown by the white dashed circle in UBM images (**e**, **f**)

Unclear scleral spur, thin iris, wide anterior chamber angle, deep anterior chamber, rarefied ciliary body, and elongated ciliary processes were observed in most PCG eyes. Abnormal anterior iris insertion was seen in 133 eyes (86.4%). Among them, 18 eyes (11.7%) had an iris insertion anterior to the scleral spur, 17 eyes (11.0%) had an anterior insertion of the iris on the scleral spur, and 98 eyes (63.6%) had a relatively anterior insertion of the iris with the iris root located posteriorly next to the scleral spur. The anterior segment photographs and UBM images of a patient with unilateral PCG are shown in Fig. 2.

### Quantitative analysis of anterior chamber parameters by UBM

As shown in Table 1, IT1 was significantly thinner in the PCG group compared to the control group (*P* < 0.001). IT1 in the PCG group ranged between 0.11 and 0.53 mm with a median of 0.35 mm, while in the control group, it ranged between 0.30 and 0.64 mm with a median of 0.44 mm. Similarly, IT2, IT3, and IT4 in the PCG group were significantly thinner than those in the control group (all *P* < 0.001). ACD and PD were found to be significantly larger in the PCG group than those in the control group (both *P* < 0.001). ACD in the PCG group ranged between 2.16 and 5.19 mm with a median of 3.77 mm, while in the control group, it ranged between 1.49 and 3.42 mm with a median of 2.82 mm. Meanwhile, eyes with PCG had a larger PD with a median of 2.35 mm compared to eyes in the control group with a median of 1.75 mm.

### Correlation of anterior chamber parameters and clinical features in PCG eyes

The correlations between the anterior chamber parameters and clinical features revealed by univariate and multivariate analyses are shown in Table 2. In univariate analysis, all the ITs and ACD were found positively correlated with age at presentation, while PD was negatively correlated with age at presentation. Bilateral PCG was significantly correlated with thinner ITs, and female gender was significantly correlated with thinner IT1. IT1 and IT2 had a significant positive correlation with AL, and IT3 had a significant negative correlation with HCD. ACD was found positively correlated with HCD, VCDR, and AL. A positive correlation was also found between PD and IOP.

**Table 2:** Correlations between anterior chamber parameters and clinical factors in the PCG group

	IT1		IT2		IT3		IT4		ACD		PD	
	Univariate	Multivariate	Univariate	Multivariate	Univariate	Multivariate	Univariate	Multivariate	Univariate	Multivariate	Univariate	Multivariate
Gender (male)	**r = 0.195** P = 0.036*	**β = 0.223** P = 0.029*	r = 0.125 P = 0.183	**/**	r = 0.013 P = 0.874	/	r = 0.012 P = 0.886	/	r = 0.137 P = 0.101	/	r = 0.030 P = 0.720	/
Laterality	**r = -0.272** P = 0.003*	**β = -0.214** P = 0.034*	r = -0.180 P = 0.054	/	**r = -0.244** P = 0.003*	**β = -0.204** P = 0.011*	**r = -0.278** P = 0.001*	**β = -0.230** P = 0.003*	r = -0.085 P = 0.310	/	r = 0.131 P = 0.113	/
Age at presentation	**r = 0.484** P < 0.001*	**β = 0.243** P = 0.036*	**r = 0.513** P < 0.001*	**β = 0.233** P = 0.041*	**r = 0.377** P < 0.001*	**β = 0.231** P = 0.004*	**r = 0.403** P < 0.001*	**β = 0.323** P < 0.001*	**r = 0.415** P < 0.001*	β = 0.151 P = 0.067	**r = -0.264** P = 0.001*	**β = -0.170** P = 0.030*
IOP	r = -0.005 P = 0.955	**/**	r = -0.027 P = 0.773	**/**	r = -0.058 P = 0.483	**/**	r = 0.028 P = 0.735	**/**	r = 0.002 P = 0.985	**/**	**r = 0.254** P = 0.002*	**β = 0.400** P < 0.001*
HCD	r = -0.081 P = 0.391	**/**	r = -0.029 P = 0.760	**/**	**r = -0.200** P = 0.015*	**β = -0.237** P = 0.003*	r = -0.072 P = 0.389	**/**	**r = 0.638** P < 0.001*	**β = 0.613** P < 0.001*	r = 0.047 P = 0.573	**/**
VCDR	r = 0.010 P = 0.932	**/**	r = 0.012 P = 0.920	**/**	r = -0.020 P = 0.842	**/**	r = 0.077 P = 0.447	**/**	**r = 0.299** P = 0.003*	β = -0.018 P = 0.822	r = 0.074 P = 0.467	/
AL	**r = 0.232** P = 0.027*	β = 0.061 P = 0.597	**r = 0.326** P = 0.002*	β = 0.223 P = 0.050	r = 0.036 P = 0.706	**/**	r = 0.178 P = 0.059	**/**	**r = 0.627** P < 0.001*	**β = 0.275** P = 0.002*	r = -0.115 P = 0.223	/

*Statistical significance; *r*, correlation coefficient; *β*, standardized coefficient; *PCG*, primary congenital glaucoma; *IT*, iris thickness; *ACD*, anterior chamber depth; *PD*, pupil diameter; *IOP*, intraocular pressure; *HCD*, horizontal corneal diameter; *VCDR*, vertical cup-to-disc ratio; *AL*, axial length

Multivariate linear regression analysis showed that thinner ITs were significantly correlated with bilateral involvement and earlier age at presentation. Additionally, thinner IT1 had a significant correlation with female gender (*P* = 0.029), and IT3 was significantly negatively correlated with HCD (*P* = 0.003). However, no correlation was found between all the IT parameters with IOP, VCDR, and AL. ACD had a positive correlation with HCD and AL (both *P* < 0.005). PD had a negative correlation with age at presentation (*P* = 0.030) and a positive correlation with IOP (*P* < 0.001).

### Risk factors of surgical failure

In the prognosis analysis, ten eyes were excluded as the postoperative follow-up time was less than 12 months. In the remaining 144 eyes, 97 eyes (67.4%) were classified as surgical success and 47 eyes (32.6%) as surgical failure. Table 3 shows both the univariate and multivariate analyses on the association of all the clinical factors and UBM parameters with surgical failure. According to the univariate logistic regression analysis, thinner IT1 (*P* = 0.003), thinner IT2 (*P* = 0.001), thinner IT3 (*P* = 0.008), and larger PD (*P* = 0.004) were associated with surgical failure. These variables with *P* < 0.05 were then entered into the multivariate logistic regression analysis to determine independent risk factors. IT1 and IT3 were excluded from the analysis since they had strong collinearity with IT2. Finally, thinner IT2 (*P* = 0.046) and larger PD (*P* = 0.049) were significantly associated with surgical failure.

**Table 3:** Correlations between risk factors and surgical failure in the PCG group

Factor	Odds ratio	95% CI	P (univariate)	P (multivariate)
Gender (male)	0.641	0.316-1.301	0.217	/
Laterality	1.726	0.820-3.631	0.148	/
Age at presentation	1.005	0.987-1.023	0.603	/
IOP	1.028	0.986-1.072	0.194	/
HCD	0.863	0.575-1.296	0.477	/
VCDR	5.313	0.160-176.357	0.345	/
AL	1.056	0.875-1.274	0.570	/
IT1	0.002	0.000-0.116	0.003*	/
IT2	0.000	0.000-0.012	0.001*	0.046*
IT3	0.000	0.000-0.048	0.008*	/
IT4	0.000	0.000-5.131	0.091	/
ACD	0.888	0.457-1.723	0.724	/
PD	1.680	1.177-2.398	0.004*	0.049*

*Statistical significance; *PCG*, primary congenital glaucoma; *IOP*, intraocular pressure; *HCD*, horizontal corneal diameter; *VCDR*, vertical cup-to-disc ratio; *AL*, axial length; *IT*, iris thickness; *ACD*, anterior chamber depth; *PD*, pupil diameter

## Discussion

UBM is useful in providing information of the structural changes in the anterior segment and is helpful for understanding the pathogenesis of PCG [11–13]. There have been several researches studying the anterior segment changes of the PCG eyes using UBM or anterior segment optical coherence tomography [11–13, 15, 16]. In this study, we quantitatively evaluated the anterior segment structures by UBM and compared these anterior chamber parameters between the PCG eyes and fellow unaffected eyes. Furthermore, we assessed the correlation between the anterior chamber parameters with disease severity and surgical prognosis.

With the help of high-resolution UBM, the morphological characteristics of PCG were identified here, including unclear scleral spur, thin iris, wide anterior chamber angle, deep anterior chamber, rarefied ciliary body, and elongated ciliary processes, which were in accordance with previous studies [11–13, 15, 16]. Abnormal anterior iris insertion was observed in 133 eyes (86.4%) in this study. The rate of anterior iris insertion in PCG eyes varied in the literature, which ranged from 11.9 to 100% [2, 12, 15, 16]. The wide variation in rates among different studies may be due to the different definitions of anterior iris insertion, different populations, and different age of patients.

ACD in the PCG group was found to be significantly deeper than in the control group, which was consistent with the prior study [12]. Unsurprisingly, a positive correlation was found between ACD with HCD and AL, which demonstrated that the deeper ACD in PCG eyes was probably caused by the progressive stretching of the eyeball, especially stretching of the anterior segment.

Our results showed that eyes with PCG had a significantly thinner IT than the fellow unaffected eyes, at the four points measured: 1 mm from the pupil edge, 2 mm from the pupil edge, 2 mm from the iris root, and 500 μm from the iris root. This is consistent with previous studies revealed by either UBM or anterior segment optical coherence tomography [11–13, 15, 16]. The ITs at 1 mm and 2 mm from the pupil edge were used to evaluate the responses of sphincter pupillae and dilator pupillae, respectively [17]. Our results showed that the ITs at 1 mm and 2 mm from the pupil edge were significantly thinner in PCG eyes, indicating the hypoplasia of both sphincter pupillae and dilator pupillae. These findings were in agreement with the histopathological studies, which had found the sphincter and dilator muscles to be markedly hypoplastic in PCG [12].

Several researchers have also detected a significant negative correlation between AL and HCD with IT and indicated that the iris thinning in PCG is probably due to stretching of the eyeball [12, 13, 15]. However, we performed a regression analysis and found no significant correlations between all the IT parameters with IOP, VCDR, or AL in this study. Meanwhile, only IT3 was negatively correlated with HCD. Similarly, Pilat et al. did not find any correlation between the mean IT and raised IOP or HCD [16]. It denoted that the iris thinning in PCG might not be the result of stretching of the eye, but be part of the primary maldevelopment of anterior chamber. We also found that younger age at presentation was associated with thinner ITs at all the measured points, which suggested that eyes with severe iris dysgenesis were prone to have an early onset. Since the IT increased with age in normal children [18, 19], we speculate that the positive correlation between the ITs and age at presentation in PCG eyes could be attributed partly to the less dysplasia of iris in older patients and partly to the normal development of the iris as the patient ages.

We also found that the PD was significantly larger in the PCG group than in the control group. The larger PD in eyes with PCG than fellow unaffected eyes in our study may indicate that the sphincter pupillae was more severely damaged than the dilator pupillae. So far, no studies have investigated the change of PD in PCG eyes, but in pediatric nonglaucomatous eyes, the PD was reported to increase with age [20, 21]. However, in our results, PD was negatively correlated with age, suggesting that eyes with an earlier onset might have more severely damaged sphincter pupillae and therefore have a larger PD. Moreover, we found a positive correlation between PD and IOP. We speculate that the larger PD we observed in PCG eyes may be a combined result of iris dysgenesis and elevated IOP.

Furthermore, we analyzed the correlation between clinical factors and UBM parameters with surgical outcomes and found that thinner IT2 and larger PD were significant risk factors of surgical failure. Meanwhile, thinner ITs were demonstrated to be associated with bilateral involvement and earlier age at presentation in our study, which were previously reported as significant predictors of the surgical failure in PCG [22, 23]. These results support the view that the degree of iris dysgenesis may indicate the degree of dysplasia of trabecular meshwork and Schlemm’s canal, and therefore is related to the severity of PCG, as well as the surgical outcome.

The limitations of this study include that the clinical information was incomplete for some patients due to the retrospective nature of our research. Besides, given that UBM can only be performed in cooperative children or children under general anesthesia, eyes of healthy children were not available as normal controls for comparison. Therefore, the fellow unaffected eyes of unilateral PCG were served as the control group, which in fact might be not purely normal.

In conclusion, we found UBM to be a powerful technique to evaluate the anterior segment structures of PCG. Eyes with PCG had a thinner iris, deeper ACD, and larger PD than the fellow unaffected eyes. Iris thinning might be part of the primary maldevelopment of anterior chamber rather than the result of stretching of the eye, and thinner ITs were significantly correlated with earlier age at presentation. Large PD might be a combined result of iris dysgenesis and elevated IOP. Thinner ITs and larger PD were significant risk factors of surgical failure in PCG.
